# Necrology

**Published:** 1899-11

**Authors:** 


### NECROLOGY.

DR. JOHN J. SMITH, veterinarian, a graduate of the American Veterinary College in the class of '79, died of anthrax, at his home in Chambersburg, Pa., September 7, 1899.

Dr. Smith was a martyr to his profession, and died as a result of his efforts to protect his community from a deadly disease.

A number of cattle and horses died suddenly on a farm near Chambersburg in the latter part of August. Dr. Smith was called upon for advice and assistance, and made post-mortem examinations on two of the animals. As anthrax had never before occurred in that neighborhood, this disease was not at first suspected; but the lesions indicated anthrax so strongly that some of the tissues were sent to the State Veterinarian for examination in his laboratory, where the diagnosis was made. Dr. Smith was at once notified of the result of the bacteriologist's examination, and full instructions and vaccine were sent for protecting the remaining stock on the farm. Unfortunately, Dr. Smith had become inoculated in one of his hands from the carcasses, and a local carbuncle commenced to develop on Sunday, September 3d. The disease progressed steadily, and on Thursday, September 7th, death ended the acute suffering.

Dr. Smith was a valued member of the veterinary profession in Pennsylvania. He was a member of the State Veterinary Medical Association, and very highly esteemed by the community in which he resided.

The following sketch is from *Public Opinion*, Chambersburg, September 8th:

"Dr. J. J. Smith fell a victim to the terrible disease of anthrax as a result of his attendance upon the diseased horses and cattle on the farm of Preston Berlin, an account of which appeared in these columns last week, and died last evening at five o'clock after intense suffering. His severe illness had been reported for several days, and when his death was announced it was received with expressions of the deepest sorrow in every part of the town.

"Dr. Smith's first symptoms of the disease were apparent on Sunday morning. He made known his fears to his brother, Joseph Smith, who called at his residence to have a talk, as was often his custom on Sunday mornings. His brother discerned that he was depressed about something, and was about to take his departure. 'Do not go,' said the doctor; 'stay with me. I shall not be here long." Asking with solicitude what was wrong, the doctor showed him three pimples on the back of his hand, saying they were evidences of blood-poisoning, with which he had by some means become inoculated in his handling of the stock which died of anthrax on the Berlin farm. It was in vain that his brother tried to dissuade him from what he believed was only a notion. The doctor said that he was positive about his case, knowing full well that the disease was incurable, and that he could not live many days. He then gave certain instructions to his brother, among others, that in the settlement of his estate no one should be distressed if they were unable to pay their accounts.

"Dr. Palmer was called in, and on Sunday evening performed the necessary surgical operation for the removal of the eruptions on his hand, Drs. Ramsey and Maclay being also present. The patient was made as comfortable as possible, but his condition was seen at once to be critical. There were the complete symptoms of the rapid ravages of the deadly disease, anthrax. The suffering of the patient might better be imagined by stating that anthrax is in the nature of a malignant ulcer, with intense burning, similar to a carbuncle. It is an infectious disease of cattle and sheep. It is ascribed to the presence of a rod-shaped bacterium (bacillus anthracis), the spores of which constitute the contagious matter. It may be transmitted to man by inoculation. The spleen becomes greatly enlarged and filled with bacteria. When not under the influence of narcotics Dr. Smith's sufferings were excruciating, and thus it was with him until released by death.

"Dr. Smith was a son of the late Jacob Smith, and was fifty-four years of age on the 2d of this month. He was educated in the common schools of Chambersburg. After learning stone-masonry he worked at the trade for several years, in the meantime diligently applying himself to the study of such veterinary works as were available. He then took a course in the American Veterinary College of New York City, from which institution he graduated in due time.

"Dr. Smith was regarded as one of the best read veterinary surgeons in the county. There were few cases he could not successfully diagnose. His eminence in this respect attained for him a wide-spread reputation, and as a consequence his practice was extensive.

"A man of positive convictions, he yet had respect for the opinions of others, and no one could be in his company without having been the better for such association. He was a devout member of the First Lutheran Church, his pastor testifying that he never missed a preparatory or communion service. He was, indeed, always in his place, whether at the regular means of grace on Sunday or weekly prayer-meeting. Fully conscious of his condition, he had an abiding faith in the consolations afforded by the religion he professed. One of his last requests was to once more partake of the Holy Communion, and his devoted pastor administered the sacred rite to him, in which his wife and daughter joined, on Tuesday.

"It is not in the church alone that Dr. Smith will be missed. Few men were more ardently devoted to the progress and welfare of Chambersburg. He served one or more terms in its town council. In the new water-works enterprise at Siloam he was a leading spirit. In the organization of the Good Will Fire Company he was a charter member, repeatedly its president, and at the time of his death its treasurer. For one or more terms he was a member of the Board of Directors of the Mechanics' Building and Loan Association. He was a Past Grand of Columbus Lodge, I. O. O. F., and Past Chief Patriarch of Olive Branch Encampment, I. O. O. F.

"Besides his wife, *née* Miss Emma Feldman, he is survived by an adopted daughter, Mrs. George Stahl, and these brothers and sisters: Adam, in the West; Jacob and Joseph, Chambersburg; Mrs. Daniel Stepler, Chambersburg; Mrs. Jos. Kline, Paw Paw, Va.; Mrs. Lizzie Dunkle, Denver, Colorado."

